# Red Light Represses the Photophysiology of the Scleractinian Coral *Stylophora pistillata*

**DOI:** 10.1371/journal.pone.0092781

**Published:** 2014-03-21

**Authors:** Tim Wijgerde, Anne van Melis, Catarina I. F. Silva, Miguel C. Leal, Luc Vogels, Claudia Mutter, Ronald Osinga

**Affiliations:** 1 Aquaculture and Fisheries Group, Department of Animal Sciences, Wageningen University and Research centre, Wageningen, The Netherlands; 2 Biological Oceanography, Royal Netherlands Institute for Sea Research, 't Horntje, The Netherlands; 3 Departamento de Biologia & CESAM, Universidade de Aveiro, Aveiro, Portugal; 4 Skidaway Institute of Oceanography, University of Georgia, Savannah, Georgia, United States of America; 5 Philips Lighting, BG Light Sources & Electronics LED Platform Development, Eindhoven, The Netherlands; King Abdullah University of Science and Technology, Saudi Arabia

## Abstract

Light spectrum plays a key role in the biology of symbiotic corals, with blue light resulting in higher coral growth, zooxanthellae density, chlorophyll *a* content and photosynthesis rates as compared to red light. However, it is still unclear whether these physiological processes are blue-enhanced or red-repressed. This study investigated the individual and combined effects of blue and red light on the health, zooxanthellae density, photophysiology and colouration of the scleractinian coral *Stylophora pistillata* over 6 weeks. Coral fragments were exposed to blue, red, and combined 50/50% blue red light, at two irradiance levels (128 and 256 μmol m^−2^ s^−1^). Light spectrum affected the health/survival, zooxanthellae density, and NDVI (a proxy for chlorophyll *a* content) of *S. pistillata*. Blue light resulted in highest survival rates, whereas red light resulted in low survival at 256 μmol m^−2^ s^−1^. Blue light also resulted in higher zooxanthellae densities compared to red light at 256 μmol m^−2^ s^−1^, and a higher NDVI compared to red and combined blue red light. Overall, our results suggest that red light negatively affects the health, survival, symbiont density and NDVI of *S. pistillata*, with a dominance of red over blue light for NDVI.

## Introduction

Light plays a key role in the growth, reproduction and physiology of scleractinian corals that host phototrophic symbionts [1], [2], [3], [4]. Until now, most studies on the effects of light on zooxanthellate corals have focused on the quantitative role of irradiance within the visible light spectrum [reviewed by 1]. In contrast, only few studies investigated the individual roles played by different colours within the visible light spectrum [5], [6], [7], [8]. It is known that not all wavelengths are equally used by different symbiotic coral species, which is associated with ecophysiological differences among coral and symbiont species [9], [10] and with selective absorption of visible light by seawater [8]. Absorption is greatest for the long wavelengths (e.g. red [8]) and, therefore, shorter wavelengths (e.g. blue) penetrate deeper into the seawater column and increase in relative proportion with depth. Blue light plays a key role in coral growth, colouration, and photophysiology, promoting coral and zooxanthellae growth, chlorophyll *a* content (either through increased zooxanthellae density or higher chlorophyll *a* per zooxanthella), fluorescent protein production, and increased photosynthesis rates [5], [6], [7], [8]. Recently, Wang et al. [11] studied the role of light spectrum on the growth and photobiology of *ex hospite* zooxanthellae (*Symbiodinium* sp., clade B), and found that blue light is essential to maintain the cell cycle and growth of these dinoflagellates. Red and infrared light resulted in little to no mitotic division of the *Symbiodinium* sp. used, respectively. Although the studies of Kinzie et al. [5] and Wang et al. [11] show that coral and zooxanthellae growth are blue-enhanced, it is still unclear whether red light acts neutrally on inhibitory on coral growth, zooxanthellae density, and photophysiology.

To address the question whether red light acts neutrally or repressively on coral photophysiology, this study investigated the individual and combined effects of narrow-bandwidth blue and red light on the health, zooxanthellae density and photophysiology of the scleractinian coral *Stylophora pistillata*. In addition, we determined how these light regimes affected the overall colouration of this species, as D'Angelo et al. [7] found that the production of colourful fluorescent host pigments, possibly acting as photoprotectants and antioxidants [12], [13], [14], is enhanced by blue light. We exposed *S. pistillata* fragments to narrow-bandwidth blue and red light, and a combination of the two, at two irradiance levels (128 and 256 μmol m^−2^ s^−1^). These irradiance levels represent the amount of blue and red irradiance found within in the first 10 meters of the seawater column, based on a photosynthetic photon flux of 2,000 μmol m^−2^ s^−1^ at sea level [15], [16] and seawater light attenuation [8]. A full spectrum light source was also included as a control to allow for comparison with previous studies [4], [17]. The findings of this study contribute to our understanding of the interplay between blue and red light on coral photophysiology. In addition, our findings may benefit sustainable coral aquaculture, which is reliant upon attractive colouration and reduced culture costs of captive-bred corals [4], [17], [18].

## Materials and Methods

### Ethics Statement

Captive-bred corals were obtained from Burgers' Zoo BV (Arnhem, The Netherlands). The experiment was conducted at Wageningen University (Wageningen, The Netherlands), with permission from Burgers' Zoo BV. No approval from an ethics committee was required as scleractinian corals are exempted from legislation concerning the use of laboratory animals in the European Union (Directive 2010/63/EU).

### Coral Fragmentation and Husbandry

**Figure 1:**
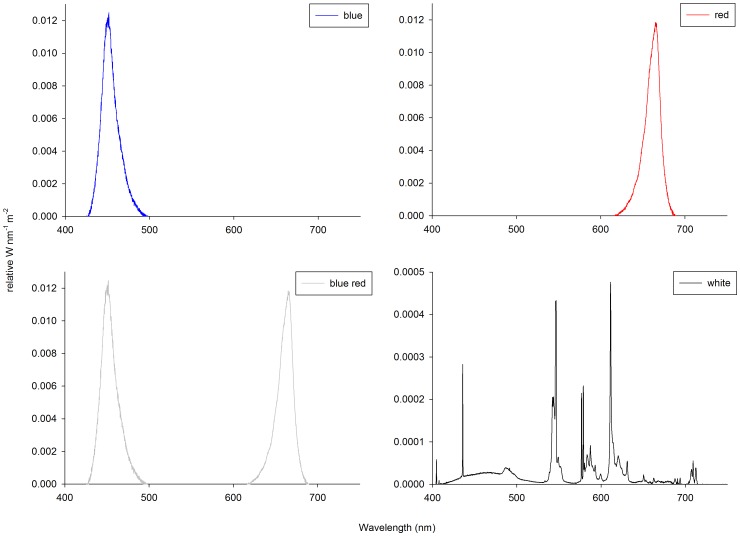
Spectral analysis of the LED and T5 fixtures used for the experiment, representative for both irradiance levels used.

The Indo-Pacific scleractinian coral *Stylophora pistillata* (Esper 1797) was used in this study. Coral fragments (*N* = 70) were randomly cut from several randomly selected colonies (all of identical genetic origin) and vertically glued onto 5×5 cm PVC tiles (Wageningen UR, Wageningen, The Netherlands) using cyanoacrylate (Gamma BV, Wageningen, The Netherlands). Only the growing tips were cut, resulting in uniform fragments roughly 1 cm in length. All fragments were allowed to recover for 7 weeks in a 400 L holding aquarium before the onset of the 6-week experiment. The holding aquarium was provided with full spectrum white light (Fig. 1), at an irradiance of 190 μmol m^−2^ s^−1^ (12 h:12 h light:dark regime), created by two 4x54W T5 fixtures (Elke Müller Aquarientechnik, Hamm, Germany). Water flow was provided by one Turbelle nanostream 6085 circulation pump (Tunze Aquarientechnik GmbH, Penzberg, Germany) providing a total flow rate of 8,000 L h^−1^. The parent colonies, which were all of the same genotype, were previously cultured for approximately 5 years at Wageningen UR under similar conditions after being obtained from Burgers' Zoo.

In the experimental system (water volume approximately 3,000 L), water flow was provided by four Turbelle nanostream 6085 circulation pumps (Tunze Aquarientechnik GmbH, Penzberg, Germany) providing a total flow rate of 32,000 L h^−1^. Water flow rate around the corals was measured with a current velocity meter (Model 2100, Swoffer Instruments, Inc., Seattle, USA) in 10 cm intervals for each experimental group, and ranged between 10 and 13 cm s^−1^ on average. The system was equipped with a MCE 600 foam fractionator (D-D The Aquarium Solution Ltd., Ilford, UK) and a 20 W UVC-light (Aqua Holland, Dordrecht, The Netherlands) powered by a 1,000 L h^−1^ aquarium pump (Eheim GmbH & Co. KG, Deizisau, Germany) to maintain water quality and clarity [19]. Constant salinity was ensured by a float sensor (Aqua Holland, Dordrecht, The Netherlands) connected to a 1,000 L h^−1^ aquarium pump (Eheim GmbH & Co. KG, Deizisau, Germany), which supplied deionised water from a 90 L holding tank. The corals were fed with 25 ml of *Artemia* nauplii suspension (approximately 3,000 nauplii mL^−1^) twice a week. Coral PVC plates were kept free of algae by biweekly cleaning with a small brush in a bucket of system water. Water parameters were maintained at the following levels: salinity 35.2±0.2 g L^−1^, temperature 26.0±0.4°C, pH 8.2±0.3, ammonium-N 0.01±0.01 mg L^−1^, nitrate-N 0.30±0.05 mg L^−1^, phosphate-P 0.28±0.03 mg L^−1^, calcium 378±39 mg L^−1^, alkalinity 3.49±0.34 mEq L^−1^ (*N* = 2-18). Trace elements were measured once with inductively coupled plasma mass spectrometry (ICP-MS), after which the following concentrations were obtained; manganese 1.41 μg L^−1^, zinc 79.70 μg L^−1^, cadmium <0.6 μg L^−1^, cobalt <0.5 μg L^−1^, chromium <0.5 μg L^−1^, copper <3.0 μg L^−1^, iron <6.0 μg L^−1^, nickel <1.2 μg L^−1^ and lead <4.0 μg L^−1^.

### Light Treatments

After the recovery period, fragments were randomly assigned to seven different light treatments (*N* = 10 per treatment); blue, red, and 50/50% blue red light, provided at a total irradiance of 128 and 256 μmol m^−2^ s^−1^ each, and full-spectrum white light at 128 μmol m^−2^ s^−1^ (Fig. 1). A 12 h:12 h light:dark regime was used for all treatments. The red, blue and 50/50% blue red light treatments were created with six custom-built 120–168W LED fixtures (Philips NV, Eindhoven, The Netherlands). To obtain two irradiance levels, three out of the six LED fixtures were dimmed using custom-built software (Philips NV, Eindhoven, The Netherlands). Full spectrum white light, created by one 4x80W T5 fixture (Elke Müller Aquarientechnik, Hamm, Germany) was included as a control, the supplied light spectrum being identical to that of the holding system. The corals, with their PVC tiles, were placed in PVC holding plates (Wageningen UR, Wageningen, The Netherlands), which in turn were placed on stainless steel tables with a seawater-proof black coating (Wageningen UR, Wageningen, The Netherlands). After positioning the tables, the corals resided at a depth of 43 cm (experimental treatments) and 74 cm (control group), respectively. To ensure equal light and flow regimes for all corals within each treatment, fragments were rotated within their holding plates twice a week during the entire experimental period.

Irradiance level (based on the photosynthetically active spectrum region or PAR, ∼400–700 nm) was measured *in situ* around the corals in the experimental tank, at 10 cm space intervals for each group, using a LI-COR 192SA quantum underwater sensor (LI-COR, Lincoln, USA). Irradiance levels were adjusted to either 128 or 256 μmol m^−2^ s^−1^ for each group except the control, for which only 128 μmol m^−2^ s^−1^ was used. The light spectra provided by the LED and T5 fixtures were determined with a calibrated HR4000 spectrometer (Ocean Optics, Dunedin, USA), which measures light in a 380–780 nm spectral range (Fig. 1). The blue LED fixture (168 W) emitted a light spectrum with a peak at 452 nm (73 nm bandwidth) and the red LED fixture (120 W) showed a peak at 665 nm (74 nm bandwidth). The 50/50% blue red LED fixture (144 W) showed a combination of the blue and red spectra (Fig. 1). The T5 full spectrum control light exhibited various peaks across the visible spectrum. Most notably, the control light emitted a significant amount of blue light, with a blue to red ratio of approximately 3. A spectral analysis was conducted for each of the two irradiance intervals applied, which revealed that spectrum was not affected by irradiance level.

### Zooxanthellae Density

Zooxanthellae density of *S. pistillata* fragments was determined after six weeks exposure to the light treatments. Four fragments from each treatment were randomly selected and cut from their PVC plates. These were subsequently weighed using the buoyant weight technique and transferred to 50 ml tubes. Tissue was removed by leading a jetstream of pressurized air through the tubes for 1 minute. Afterwards, 10 ml of artificial seawater (ASW) was added and each tube was shaken vigorously for 3 minutes to remove all tissue from the wall of the tube and the skeleton. Each coral skeleton was removed with tweezers and the tube centrifuged for 10 minutes at 4°C and 4,000 rpm. The supernatant, containing the animal fraction, was carefully removed and the pellet, containing the heavier zooxanthellae was resuspended in 750 μL ASW. Total volume of each suspension was determined using a 1,000 μl pipette. Small volumes of homogenised samples were transferred to a Neubauer improved counting chamber (LO-Laboroptik Ltd, Lancing, UK), and zooxanthellae scored. Finally, zooxanthellae density was calculated using the pellet volume and buoyant weight of each coral fragment. The accuracy of this method was assured by using branching corals with a highly constant surface/volume and surface/mass ratio [4].

### Maximum Quantum Yield

Pulse Amplitude Modulation (PAM) fluorometry was used to non-intrusively monitor the maximum quantum yield (MQY) of photosystem II within the zooxanthellae [20]. A PAM fluorometer (Heinz Walz GmbH, Effeltrich, Germany) was used to measure MQY on two sides of each coral fragment. These measurement positions were kept constant during the experiment. All measurements were performed weekly *in situ*, 1–2 h before the start of the daylight period, to prevent photosystem activation. Measuring and saturating lights were provided by a full spectrum lamp and delivered to the sample by a 5 mm diameter plastic fibre optic cable. The fibre optic was positioned approximately 2 mm from the surface of the coral fragment, after which a saturating pulse of 1.2 s was applied to determine the minimum or dark-level fluorescence (F_0_), and maximum fluorescence (F_M_). F_0_ and F_M_ were used to determine the MQY of PSII using the following formula [20]:
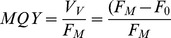



### Coral Spectral Reflectance and NDVI

Diffusive reflectance spectra were measured weekly over a 190–892 nm bandwidth, with a spectral resolution of 0.33 nm, using a USB2000 spectrometer (USB2000-VIS-NIR, grating #3, Ocean Optics, Dunedin, USA) connected to a 400 μm diameter fibre optic cable (QP400-2-VIS/NIR-BX, Ocean Optics, Dunedin, USA). To minimise background reflection, each coral fragment was removed from the aquarium and placed in a black, Teflon-coated container, filled with water from the experimental system. The fibre optic was maintained perpendicular to the coral surface, at a fixed distance, defined to match a view field covering a circular area of approximately 3 mm diameter on the surface of each coral fragment. During measurements, the coral fragments and the reference white panel (see below) were measured under a full spectrum halogen light (Philips, Eindhoven, The Netherlands), aimed at an approximate 45° angle to the table. The light spectrum reflected from each coral fragment was normalised to the spectrum reflected from a white reference standard (WS-1-SL Spectralon Reference Standard, Ocean Optics, Dunedin, USA). The reflectance spectrum measured in the dark was subtracted from both spectra to account for the dark current noise of the spectrometer. All coral fragments were measured on four different sides each. These measurement positions were kept constant during the experiment. The four measurements were averaged before being used for subsequent calculations.

The Normalised Difference Vegetation Index (NDVI) [21] was used as a proxy for chlorophyll *a* content [17], [22] and calculated with the formula below, where R_750_ and R_675_ represent the average diffusive reflectance in the intervals of 749.73–750.39 nm and 674.87–675.55 nm, respectively.
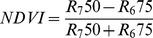



### Photographic Analysis

At the end of the experimental treatment, three corals from each experimental treatment were randomly selected for close-up photography. Each coral was placed in a 60 L aquarium with water from the experimental system (approximately 40 L), and individually photographed with a D5000 DSLR camera equipped with a Nikkor AF-D 60 mm macro lens (Nikon, Tokyo, Japan). An external SB700 flash unit (Nikon, Tokyo, Japan) was positioned approximately 30 cm above the coral for additional illumination. All corals were photographed using the same camera and flash settings, including white balance, aperture, exposure time and ISO sensitivity. A 5×5 cm PVC plate was used for scale.

### Data Analysis

Several corals from various treatments showed necrosis from week 4 onwards. To determine MQY, reflectance and NDVI of live coral tissue only, we omitted data from necrotic and dead colonies. Normality of data was tested by plotting residuals of each dataset versus predicted values, and by performing a Shapiro-Wilk test. Homogeneity of variances was determined using Levene's test. All data were found to be normally distributed and homoscedastic after a ^10^log transformation (P>0.050). We used a two-way factorial ANOVA to test the (interactive) effects of spectrum and irradiance on zooxanthellae densities, MQY and NDVI, and a mixed factorial ANOVA to test for differences in MQY between week 1 and 6. A Bonferroni *post-hoc* test was used to determine differences between spectrum levels. Simple effects analysis was employed to break down interactive effects. A *P*<0.050 value was considered statistically significant. Statistical analysis was performed with SPSS Statistics 20 (IBM, Somers, USA). Graphs were plotted with SigmaPlot 12 (Systat software, San Jose, USA). All data presented are expressed as means + standard deviation, unless stated otherwise.

## Results

### Coral Health and Survival

**Figure 2:**
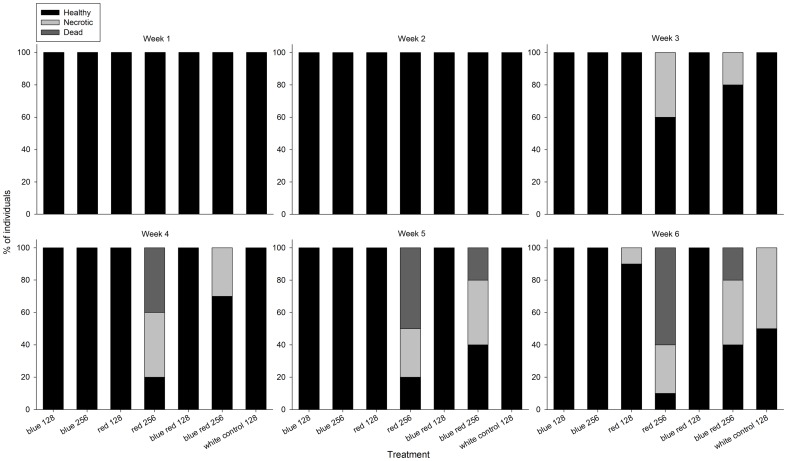
Coral health and survival under various experimental conditions (blue, red, 50/50% blue red and white light at an irradiance of 128/256 μmol m^−2^ s^−1^) over a time course of 6 weeks (*N* = 10).

During the experiment, corals exhibited different health and survival patterns between treatments (Fig. 2). After week 3, corals grown under red and blue red light at an irradiance of 256 μmol m^−2^ s^−1^ (red 256 and blue red 256) started to show necrosis, which continued to progress towards mortality after week 4 and beyond. In addition, corals grown under red and white control light at an irradiance of 128 μmol m^−2^ s^−1^ (red 128 and white control 128) exhibited necrosis at the end of the experiment. Corals maintained under blue light exhibited no necrosis or mortality, regardless of irradiance.

### Zooxanthellae Density

**Figure 3:**
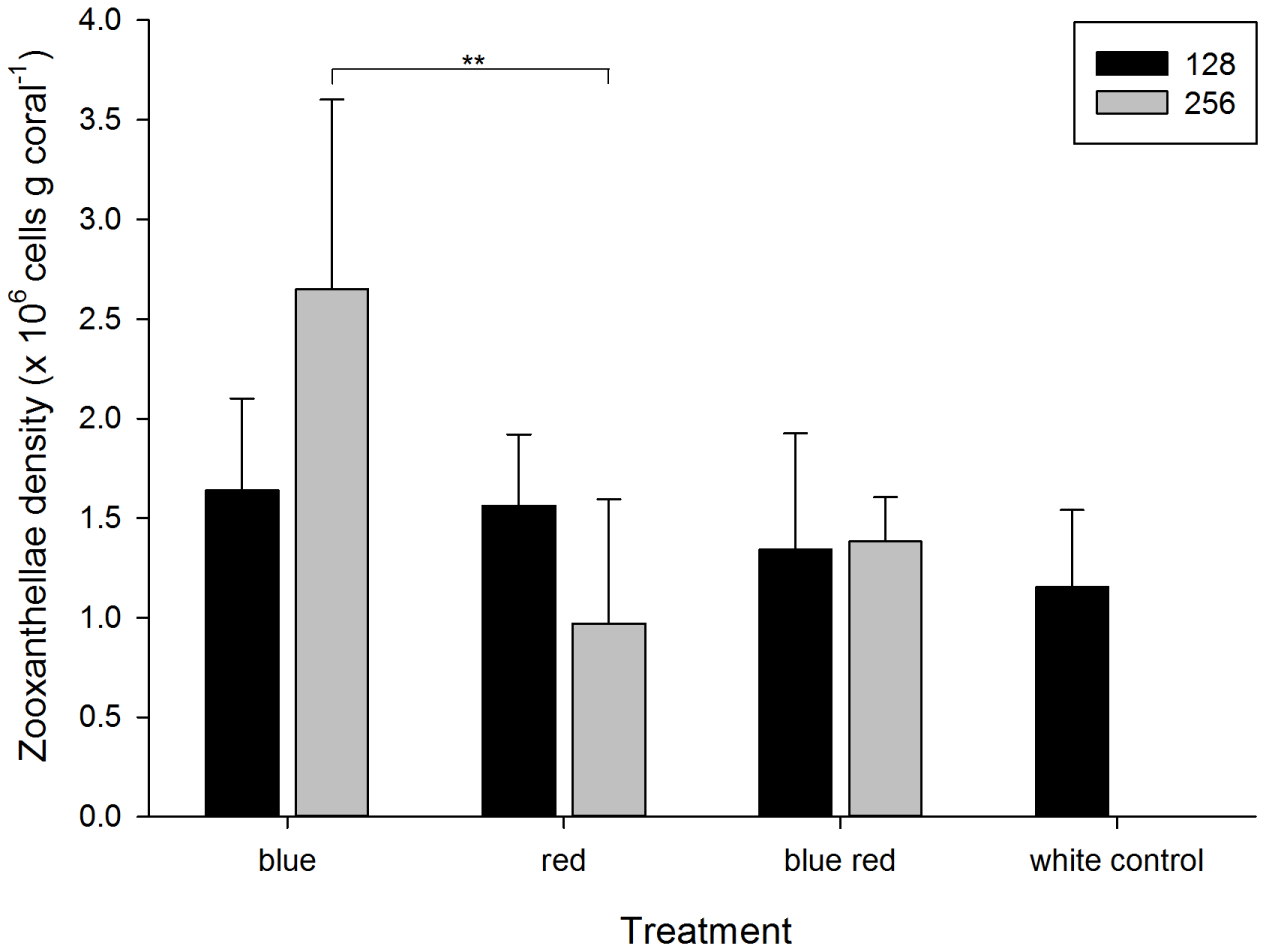
Zooxanthellae density under various experimental conditions (blue, red, 50/50% blue red and white light at an irradiance of 128/256 μmol m^−2^ s^−1^) after week 6. Values are means + s.d. (*N* = 4). **Indicates significant difference (*P*<0.010).

Zooxanthellae density at the end of week 6 ranged from 0.97±0.62×10^6^ to 2.65±0.95×10^6^ cells per gram coral (Fig. 3) and was significantly affected by light spectrum (Table 1). In addition, an interactive trend of spectrum and irradiance was detected (Table 1), with the effect of spectrum on zooxanthellae density observed for corals grown under an irradiance level of 256 μmol m^−2^ s^−1^ (F_2,16_ = 6.952, *P* = 0.007). Specifically, corals grown under a blue irradiance of 256 μmol m^−2^ s^−1^ (blue 256) showed a significantly higher zooxanthellae density compared to those cultured under red light at the same irradiance (red 256, *P* = 0.006). There was also a positive trend for the effect of blue irradiance on zooxanthellae density, with a potentially higher zooxanthellae density under blue light at an irradiance of 256 μmol m^−2^ s^−1^ (blue 256) compared to 128 μmol m^−2^ s^−1^ (blue 128, *P* = 0.122). For red light, the opposite trend was observed, i.e. a potentially lower zooxanthellae density for corals exposed to red light at an irradiance of 256 μmol m^−2^ s^−1^ (red 256) as compared to 128 μmol m^−2^ s^−1^ (red 128, *P* = 0.072).

**Table 1:** Two-way factorial ANOVA, demonstrating main and interactive effects of spectrum and irradiance on zooxanthellae density, maximum quantum yield (MQY) and NDVI (*N* = 1-10).

Factor	Variable	F	df	error	*P*
	Zooxanthellae density				
Spectrum		4.133	2	16	0.036*
Irradiance		0.024	1	16	0.878
Spectrum * Irradiance		3.222	2	16	0.067
	MQY week 1				
Spectrum		0.516	2	54	0.600
Irradiance		0.584	1	54	0.448
Spectrum * Irradiance		2.704	2	54	0.076
	MQY week 2				
Spectrum		1.357	2	54	0.266
Irradiance		0.203	1	54	0.654
Spectrum * Irradiance		2.000	2	54	0.145
	MQY week 3				
Spectrum		1.363	2	54	0.265
Irradiance		0.497	1	54	0.484
Spectrum * Irradiance		4.462	2	54	0.016*
	MQY week 4				
Spectrum		0.063	2	43	0.939
Irradiance		2.670	1	43	0.110
Spectrum * Irradiance		0.120	2	43	0.887
	MQY week 5				
Spectrum		0.364	2	40	0.697
Irradiance		4.670	1	40	0.037*
Spectrum * Irradiance		1.212	2	40	0.308
	MQY week 6				
Spectrum		1.467	2	33	0.245
Irradiance		6.090	1	33	0.019*
Spectrum * Irradiance		0.109	2	33	0.897
	NDVI week 1				
Spectrum		0.026	2	54	0.975
Irradiance		0.683	1	54	0.412
Spectrum * Irradiance		5.814	2	54	0.005*
	NDVI week 2				
Spectrum		4.585	2	54	0.014*
Irradiance		0.330	1	54	0.568
Spectrum * Irradiance		1.423	2	54	0.250
	NDVI week 3				
Spectrum		1.775	2	54	0.179
Irradiance		7.789	1	54	0.007*
Spectrum * Irradiance		2.229	2	54	0.117
	NDVI week 4				
Spectrum		5.458	2	43	0.008*
Irradiance		8.799	1	43	0.005*
Spectrum * Irradiance		7.296	2	43	0.002*
	NDVI week 5				
Spectrum		3.495	2	38	0.040*
Irradiance		0.368	1	38	0.548
Spectrum * Irradiance		2.831	2	38	0.071
	NDVI week 6				
Spectrum		38.359	2	33	0.000*
Irradiance		0.702	1	33	0.408
Spectrum * Irradiance		4.588	2	33	0.017*

*Indicates significant effect (*P*<0.050).

### Maximum Quantum Yield

**Figure 4:**
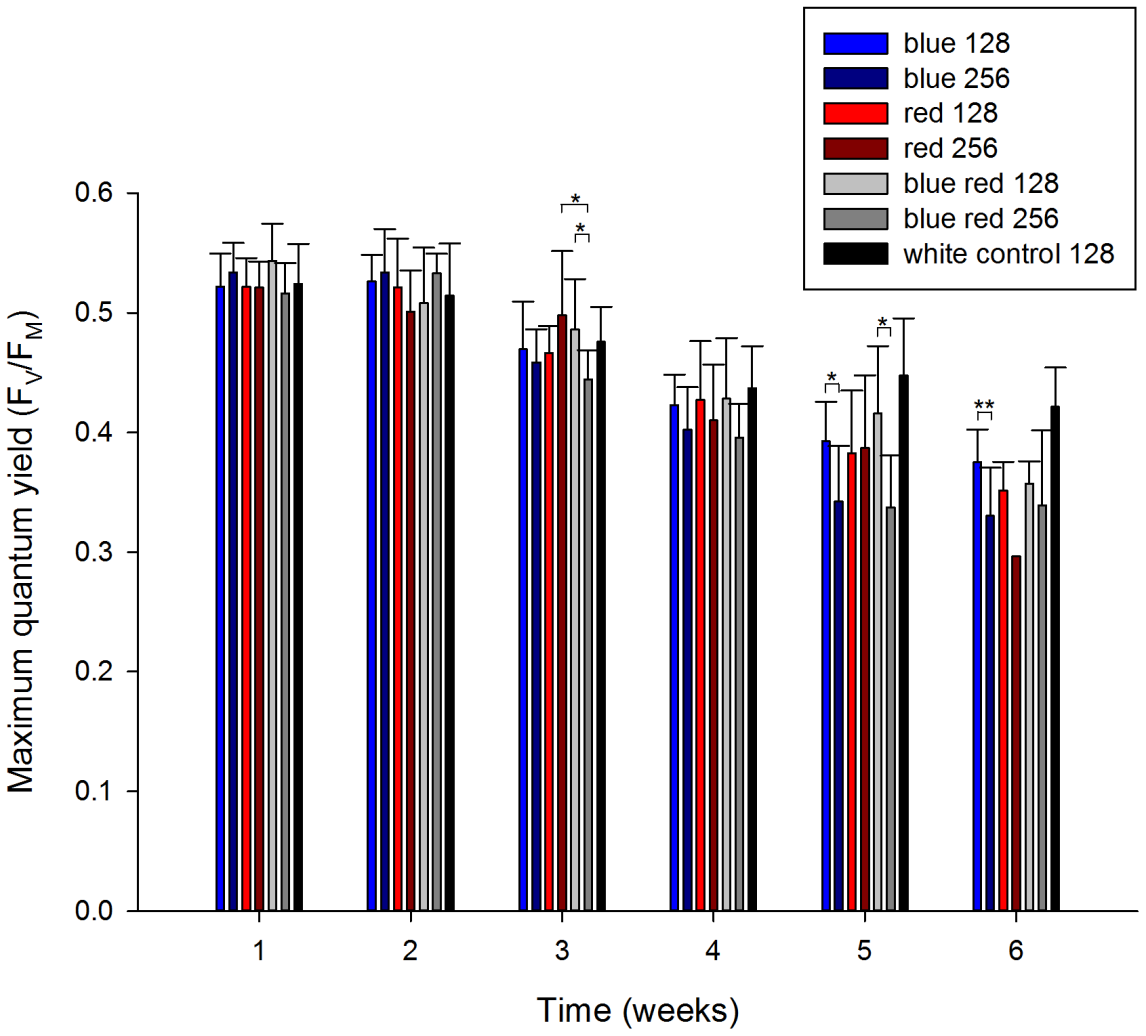
Maximum quantum yield of corals under various experimental conditions (blue, red, 50/50% blue red and white light at an irradiance of 128/256 μmol m^−2^ s^−1^) over a time course of 6 weeks. Values are means + s.d. (*N* = 1-10). *Indicates significant difference (*P*<0.050), **(*P*<0.010).

Overall, all corals showed a decreasing trend in maximum quantum yield (MQY) during the experiment (Fig. 4). After week 6, MQY was significantly lower compared to after week 1, irrespective of spectrum and irradiance (F_1,33_ = 380.073, *P* = 0.000). Significant differences in MQY between treatments were found from week 3 onwards. A significant interactive effect of spectrum and irradiance was found for MQY after week 3 (Table 1) as revealed by higher MQY for corals grown under red 256 compared to blue red 256 (*P* = 0.010), and higher for corals grown under blue red 128 compared to blue red 256 (*P* = 0.020). After week 5, a significant effect of irradiance was detected (Table 1), with corals grown under blue 128 displaying a higher MQY compared to those cultured under blue 256 (*P* = 0.035). The same trend between irradiance levels was observed for the blue red treatments (*P* = 0.021). After week 6, a significant effect of irradiance was detected (Table 1), with corals cultured under blue 128 exhibiting a higher MQY than those exposed to blue 256 (*P* = 0.009).

### Normalised Difference Vegetation Index (NDVI)

**Figure 5:**
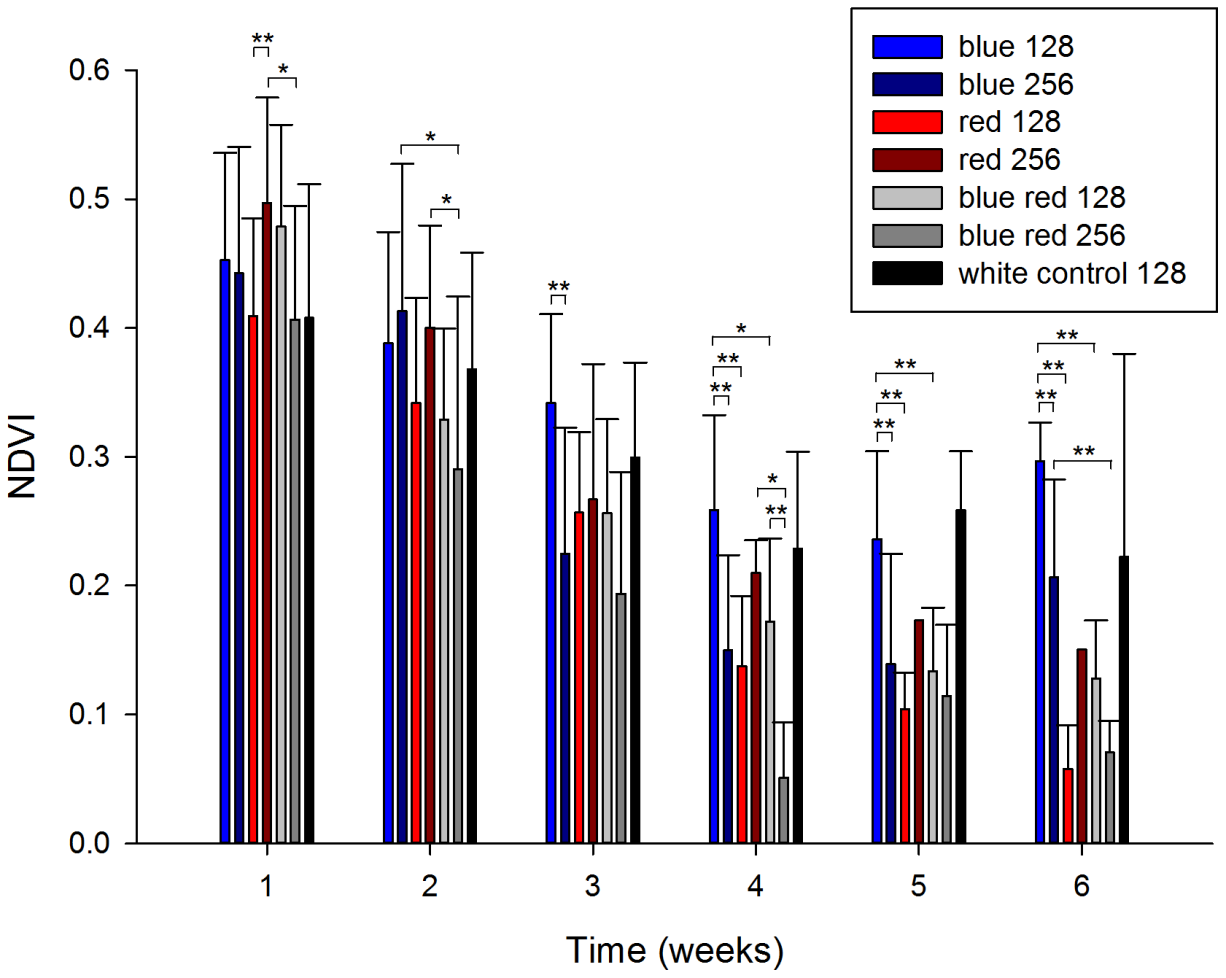
Normalised Difference Vegetation Index (NDVI) of corals under various experimental conditions (blue, red, 50/50% blue red and white light at an irradiance of 128/256 μmol m^−2^ s^−1^) over a time course of 6 weeks. Values are means + s.d. (*N* = 1-10). *Indicates significant difference (*P*<0.050), **(*P*<0.010).

Similar to MQY, most corals exhibited a decreasing NDVI trend during the experiment, with corals exposed to blue 128 showing the highest value at the end of the experiment (Fig. 5). A significant interactive effect of spectrum and irradiance on NDVI was found at week 1 (Table 1), with corals grown under red 256 exhibiting a higher NDVI compared to those from the blue red 256 (*P* = 0.040) and red 128 treatments (*P* = 0.004). After week 2, a main effect of spectrum was found (Table 1), with corals grown under blue 256 and red 256 showing a higher NDVI compared to corals maintained under blue red 256 (*P* = 0.021 and *P* = 0.029, respectively). After week 3, a main effect of irradiance was detected (Table 1), with a higher NDVI for corals cultured under blue 128 compared to those kept under blue 256 (*P* = 0.004). After week 4, significant main and interactive effects of spectrum and irradiance were found (Table 1). Specifically, corals grown under blue 128 showed a higher NDVI compared to those cultured under blue 256, red 128, and blue red 128 (*P* = 0.000, *P* = 0.000 and *P* = 0.013, respectively). In addition, corals grown under red 256 and blue red 128 had a higher NDVI values than those kept under blue red 256 (*P* = 0.001 and *P* = 0.018, respectively). After week 5, a significant effect of spectrum was detected (Table 1), with corals cultured under blue 128 exhibiting a higher NDVI than those grown under blue 256 (*P* = 0.002), red 128 (*P* = 0.000), and blue red 128 (*P* = 0.004). After week 6, main and interactive effects of spectrum were found (Table 1), with a higher NDVI for corals maintained under blue 128 compared to blue 256 (*P* = 0.000), red 128 (*P* = 0.000) and blue red 128 (*P* = 0.000), and a higher NDVI for corals kept under blue 256 as compared to those grown under blue red 256 (*P* = 0.000).

### Reflectance

**Figure 6:**
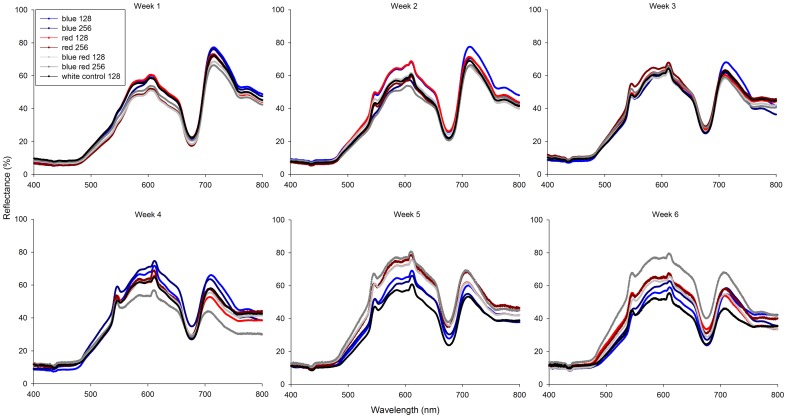
Coral reflectance under various experimental conditions (blue, red, 50/50% blue red and white light at an irradiance of 128/256 μmol m^−2^ s^−1^) over a time course of 6 weeks. Values are means (*N* = 1-10).

Coral reflectance changed during the experimental period (Fig. 6). After week 2 and beyond, corals grown under all treatments exhibited small, but distinct reflectance peaks at 545 and 611 nm. These peaks reached their maximum after week 2 for red 128, week 4 for the blue treatments and week 5 for the red and blue red 256 treatments. In addition, all corals clearly showed reflection minima at wavelengths below 500 and at 670 nm. Moreover, after week 6, corals exposed to blue red 256 reflected more light in the 480–750 nm range, with the most pronounced changes in the green/yellow/orange/red part of the light spectrum (540–650 nm). Finally, the reflectance amplitude between the minimum at 670 nm and the maximum at 750 nm decreased over time for all treatments.

### Colouration

**Figure 7:**
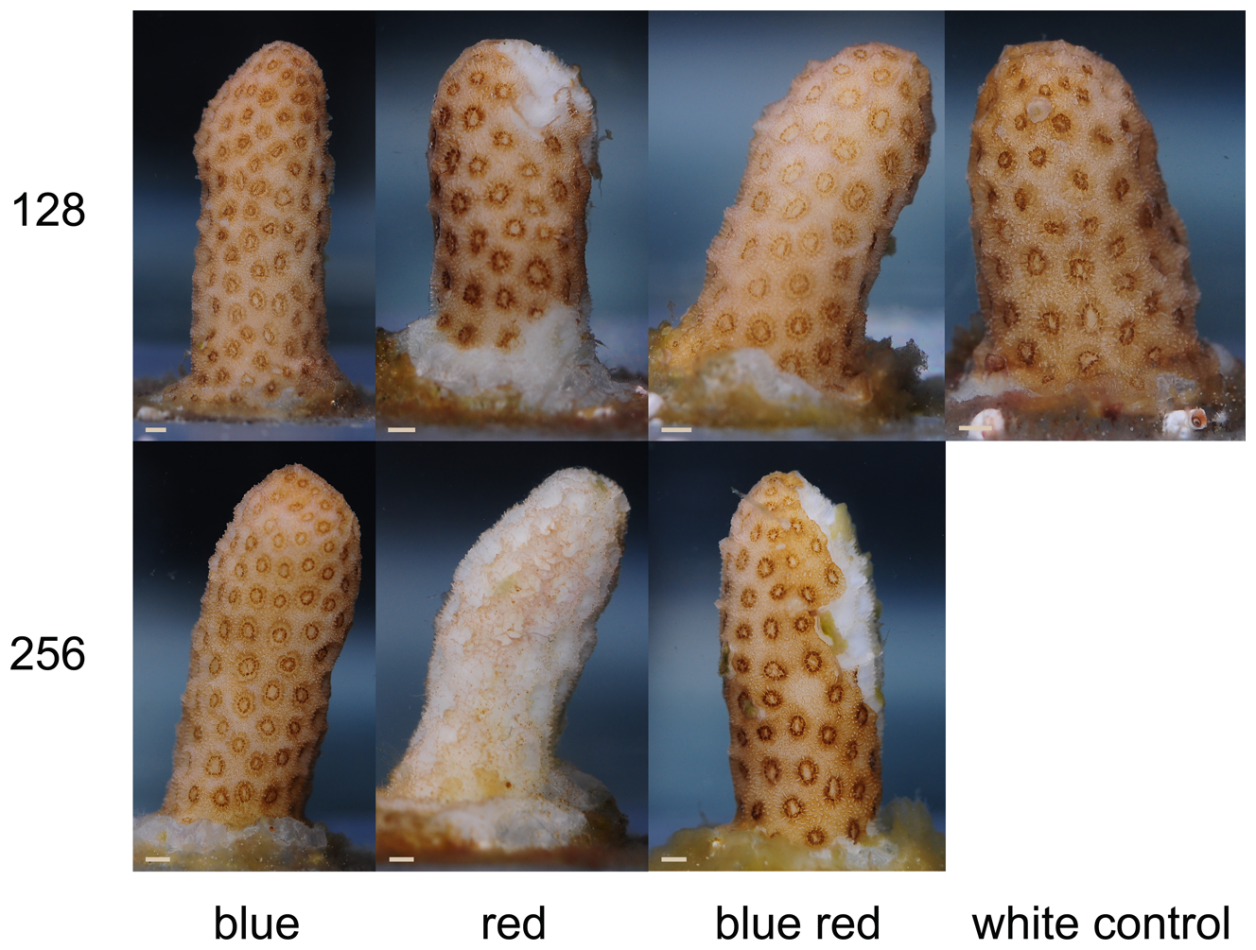
Close-up images of corals grown under various experimental conditions (blue, red, 50/50% blue red and white light at an irradiance of 128/256 μmol m^−2^ s^−1^) after week 6. Scale bars: 1 mm.

Corals were photographed at the end of week 6 to assess visible changes in their colouration. Corals grown under white 128, red 128 and blue red 256 showed increased pigmentation of polyp tentacles (Fig. 7). In addition, corals cultured under blue red 256 displayed a yellow/orange hue of the coenenchyme. This last feature was consistent with the reflection pattern of these corals after week 6 (Fig. 6), with a pronounced reflection increase in the green to red part of the light spectrum (550–650 nm). Despite the presence of necrotic and dead corals (Fig. 2), and low MQY values (Fig. 4) at the end of the experimental period, remaining coral tissue appeared healthy, with no signs of bleaching.

## Discussion

This study revealed distinct effects of light spectrum and irradiance on the health/survival, symbiont density, photophysiology and colouration of the scleractinian coral *Stylophora pistillata*. Most notably, red light seemed to exert an inhibitory effect on zooxanthellae density and NDVI, a proxy for chlorophyll *a* content.

Coral health and survival were markedly affected by light spectrum, with highest survival rates (100%) for corals exposed to blue light, regardless of irradiance. The only treatments that resulted in necrosis and/or mortality were those that included red light, either solely or combined with blue light. However, no adverse effect was found at a red irradiance of 64 μmol m^−2^ s^−1^ (blue red 128; Fig. 2), which suggests that red light may promote necrosis and mortality of *S. pistillata* at irradiance of 128 μmol m^−2^ s^−1^ and above (either due to additional blue or red light). Although shallow-growing corals are exposed to red light of similar intensity as used in this study [8], it is possible that the genotype used for this experiment was collected at a depth where red light is nearly or completely absent (<10 m) [8], rendering this coral sensitive to excess red light. A caveat that has to be considered here is the necrosis of corals grown under the white light, which started during week 6. As this light spectrum is known to be suitable for aquaculture of this particular genotype [4], [17], this suggests that other factors than light may have caused necrosis and mortality at the end of the experiment. A possible candidate is zinc, a co-factor of many enzymes with important roles in metabolism, and known to affect photosynthetic efficiency of *S. pistillata* [23]. More specifically, the zinc concentration in the experimental system was 79.7 μg L^−1^, a concentration known to be in the toxic range for some coral species [24]. It is thus notable that narrow-bandwidth blue light seems favourable in a high-zinc environment, although it is unclear why. It is possible that blue light protects against zinc-induced reactive oxygen species [25], [26], [27] by enhancing the production of fluorescent proteins [7], pigments that possess antioxidant activity [12], [28] (also see below on reflection).

Light spectrum also affected zooxanthellae density, which was significantly higher under blue light compared to red light at an irradiance of 256 μmol m^−2^ s^−1^. In addition, a positive, dose-dependent trend of blue light on zooxanthellae density was visible, as higher blue irradiance seemed to result in a higher zooxanthellae density. This contrasts with typical reports of increased zooxanthellae density at lower irradiance [22], [29], [30], which is observed in nature with increasing depth, where blue light is proportionally higher. Although such changes in zooxanthellae densities are usually associated with decreased irradiance, our results suggest that the increased ratio of blue to red light may also be an important regulator of zooxanthellae populations *in hospite*. As this study shows opposite trends of blue and red light, it is likely that both spectrum ranges play an active role in regulating zooxanthellae density, with blue light being stimulatory, and red light inhibitory. This theory is supported by a neutral effect on zooxanthellae density with a combined increase in blue and red light (Fig. 3), possibly because blue and red light compensated for one another's effect. Although this proposed mechanism seems to contradict the study of Kinzie et al. [5], who showed that blue and white light have similar effects on *in hospite* zooxanthellae, the white light treatment they applied exhibited higher blue than red irradiance. It is also possible that our highest blue red treatment did not emit sufficient blue and red light (128/128 μmol m^−2^ s^−1^) for these colours to have a significant positive and negative effect, respectively.

During the experimental period, all corals exhibited a decreasing trend in MQY. Although this may be indicative of stress, e.g. photoinhibition of photosystem II [31] or elevated zinc levels in the experimental system (see above), corals maintained under blue and white light showed a less pronounced negative trend in MQY. In addition, corals cultured under blue light had a significantly higher MQY at the lower irradiance applied, which suggests that higher energy associated with blue light causes (more) damage to photosystem II, possibly through D1 protein degradation [31]. At the end of the experiment, corals exposed to red 256 exhibited a MQY of 0.3 and were starting to bleach, which may have resulted in the necrosis that ensued.

Similarly to MQY, all corals showed a decreasing trend in NDVI. Although this may also be indicative of zooxanthellae expulsion associated with stress (see above), corals maintained under blue and white light showed a less pronounced NDVI decrease, especially at lower blue irradiance. This suggests that narrow-bandwidth blue and white light (with a high blue:red ratio of ∼3; Fig. 1) indeed favour chlorophyll *a* production over red light, in agreement with the findings of Kinzie et al. [5]. The question is whether differences in chlorophyll *a* synthesis are caused by an enhancement by blue light, a repression by red light, or both. The data suggest that red light actively represses chlorophyll *a* synthesis, with a dominance over blue light, as all corals exposed to a significant amount of red light (red 128/256 and blue red 128/256) exhibited low NDVI values. As zooxanthellae densities between all treatments were similar (apart from blue 256 and red 256), red light may repress chlorophyll *a* synthesis per zooxanthella in *S. pistillata*.

All corals used in this experiment displayed distinct reflectance peaks at 545 and 611 nm from week 2 onwards. This may be due to increased production of green and red fluorescent proteins, respectively [7], [32], [33], [34], which may be related to protection against zinc-induced oxygen radicals [12], [25], [26], [27], [28]. The reflection minima observed at 440 and 670 nm are probably due to the presence of chlorophyll *a*, which absorbs light at these wavelengths [35], [36].

Next to changes in reflection patterns over time, corals grown under red 128 and blue red 256 exhibited pronounced brown, pigmented tentacles, possibly associated with photopigments from zooxanthellae. This may be associated with a redistribution of zooxanthellae over time as these corals neither showed increased zooxanthellae densities nor elevated NDVI compared to other groups. Concentrated symbionts within polyp tentacles may increase self-shading of zooxanthellae, and bestow a degree of protection upon the zooxanthellae and corals by reducing D1 protein damage of photosystem II [31]. After week 6, corals cultured under blue red 256 displayed a yellow/orange hue of the coenenchyme, consistent with the reflection pattern in the green to red part of the spectrum (540–650 nm). It is unclear why this occurred, but it may reflect a higher capacity for fluorescence of UV radiation as yellow light [37], even though our experimental lights did not emit any UV. Although a bright, fluorescent colouration of captive-bred corals is important for their market value [17], our light treatments only resulted in moderate colour differences in *S. pistillata* (Figs. 6 and 7), in line with the results of [17]. This suggests that for aquaculture of this genotype, selecting the light regime resulting in an optimal ratio between growth and energy consumed can be done without having to compromise for colouration.

In conclusion, our findings suggest that red light actively represses symbiont density and NDVI, a proxy for chlorophyll *a* content, in the *ex situ* cultured coral *Stylophora pistillata*. The ecological implication is that red light may be an important sensory cue to detect high irradiance, *sensu* [5], negatively regulating zooxanthellae density and chlorophyll *a* synthesis to reduce photodamage and bleaching sensitivity. This theory is consistent with the amount of red light present at low depth (<10 m), which is similar to our highest red irradiance treatment [8], [15], [16]. The mechanisms behind the inhibitory effects of red light are possibly linked to red-sensitive phytochromes, which regulate many processes in plants including chlorophyll biosynthesis [38], [39] and which have been identified in zooxanthellae [39]. In addition, blue light is beneficial to the health, survival, symbiont density and NDVI of *S. pistillata*. This important role of blue light for the wellbeing of the coral holobiont may be based on blue-sensitive cryptochromes, which have been implicated in the regulation of circadian rhythm and sexual reproduction in corals [3], [40], [41], [42], and the cell cycle of dinoflagellates [11], [43]. Finally, our findings may benefit the sustainable aquaculture of this species, with narrow-bandwidth blue light sources seeming most suitable for this particular genotype.
